# Speaking up for the lost voices: representation and inclusion of people with communication impairment in brain tumour research

**DOI:** 10.1007/s00520-023-07804-5

**Published:** 2023-05-27

**Authors:** Fiona Menger, Harriet Cresswell, Joanne Lewis, Anna Volkmer, Linda Sharp

**Affiliations:** 1 Speech and Language Sciences, School of Education, Communication and Language Sciences, Newcastle University, Newcastle Upon Tyne, NE1 7RU UK; 2 Chesterfield Royal Hospital NHS Foundation Trust, Chesterfield, UK; 3 Newcastle Upon Tyne Hospitals NHS Foundation Trust, Newcastle Upon Tyne, UK; 4 Psychology and Language Sciences, University College London, London, UK; 5 Population Health Sciences Institute, Newcastle University, Newcastle Upon Tyne, UK

**Keywords:** Brain tumours, Quality of life, Communication impairments, Advocacy

## Abstract

Brain tumours and their associated treatments can lead to progressive impairments of communication, adversely affecting quality-of-life. This commentary explores our concerns that people with speech, language, and communication needs face barriers to representation and inclusion in brain tumour research; we then offer possible solutions to support their participation. Our main concerns are that there is currently poor recognition of the nature of communication difficulties following brain tumours, limited focus on the psychosocial impact, and lack of transparency on why people with speech, language, and communication needs were excluded from research or how they were supported to take part. We propose solutions focusing on working towards more accurate reporting of symptoms and the impact of impairment, using innovative qualitative methods to collect data on the lived experiences of speech, language, and communication needs, and empowering speech and language therapists to become part of research teams as experts and advocates for this population. These solutions would support the accurate representation and inclusion of people with communication needs after brain tumour in research, allowing healthcare professionals to learn more about their priorities and needs.

## Introduction

In 2020, an estimated 308,100 primary malignant brain tumours were diagnosed worldwide [1]. A brain tumour and the treatment thereof, can lead to progressive impairment of communication, adversely affecting interactions with others and multiple aspects of quality-of-life (QoL) [2]. Cancer survivorship research seeks to understand the experiences and impacts of cancer to inform the need for services and/or development of interventions. This commentary outlines our concerns regarding the representation and inclusion of brain tumour patients with speech, language, and communication needs (SLCN) in such research and based on our expertise, proposes ways forward.

## Concerns

### Poor recognition of the nature of SLCN following brain tumour

Speech, language, and communication impairments following brain tumours may progress slowly and be difficult to detect [3], meaning it can be challenging to capture their impact. Cognitive difficulties may also impair communication. While formal assessment is more likely to demonstrate difficulties than self-report [4], some language assessments lack sensitivity and specificity [3, 5]. People with brain injury may also have limited insight into their difficulties [6]. Despite this, scale measures frequently contain broad terms such as ‘difficulty speaking’ or ‘word finding difficulties’, which are insensitive to the precise nature of impairment. For those with more severe SLCN, researchers often rely on carer report, which may not be an accurate proxy measure [7].

### Limited understanding of the psychosocial impact

While survivorship studies on brain tumours are relatively uncommon, there is a growing focus on QoL outcomes. Within quantitative studies, communication-related measures are infrequently used [8]. Some more broadly focused qualitative studies provide a glimpse into the impact of SLCN. For example, concerns about speech [9], lack of awareness and insight [10], or the adaptations needed by carers to support communication [11, 12]. Across both qualitative and quantitative research, those with more severe language and literacy impairments may be daunted to take part or experience considerable barriers.

### Transparency around inclusion in research

Participation in research can be demanding of both communication and cognitive skills, and potential participants are commonly excluded due to reasons such as ‘unable to communicate adequately’, or ‘do not understand due to cognitive or language impairment’. In such situations, it is important to question whether excluded participants with SLCN could be facilitated to understand the nature of a study, to consent, or to participate, and whether included participants have been provided with all the help they need. Such provision can be viewed alongside relevant legislation, such as the Mental Capacity Act (2005) in the UK [13]. In the field of stroke, there have also been calls for better inclusion [14]; researchers have reported they lack tools and support to determine capacity and obtain consent when working with neurologically impaired people [15]. It is possible these same barriers exist for those working with brain tumour populations.

Failure to represent this population adequately results in a lack of evidence to support appropriate interventions towards improving functioning and QoL. There are three main areas where we believe that improvements could be made:

## Solutions

### Work towards improving accuracy of reporting of impairment

**Fig. 1:**
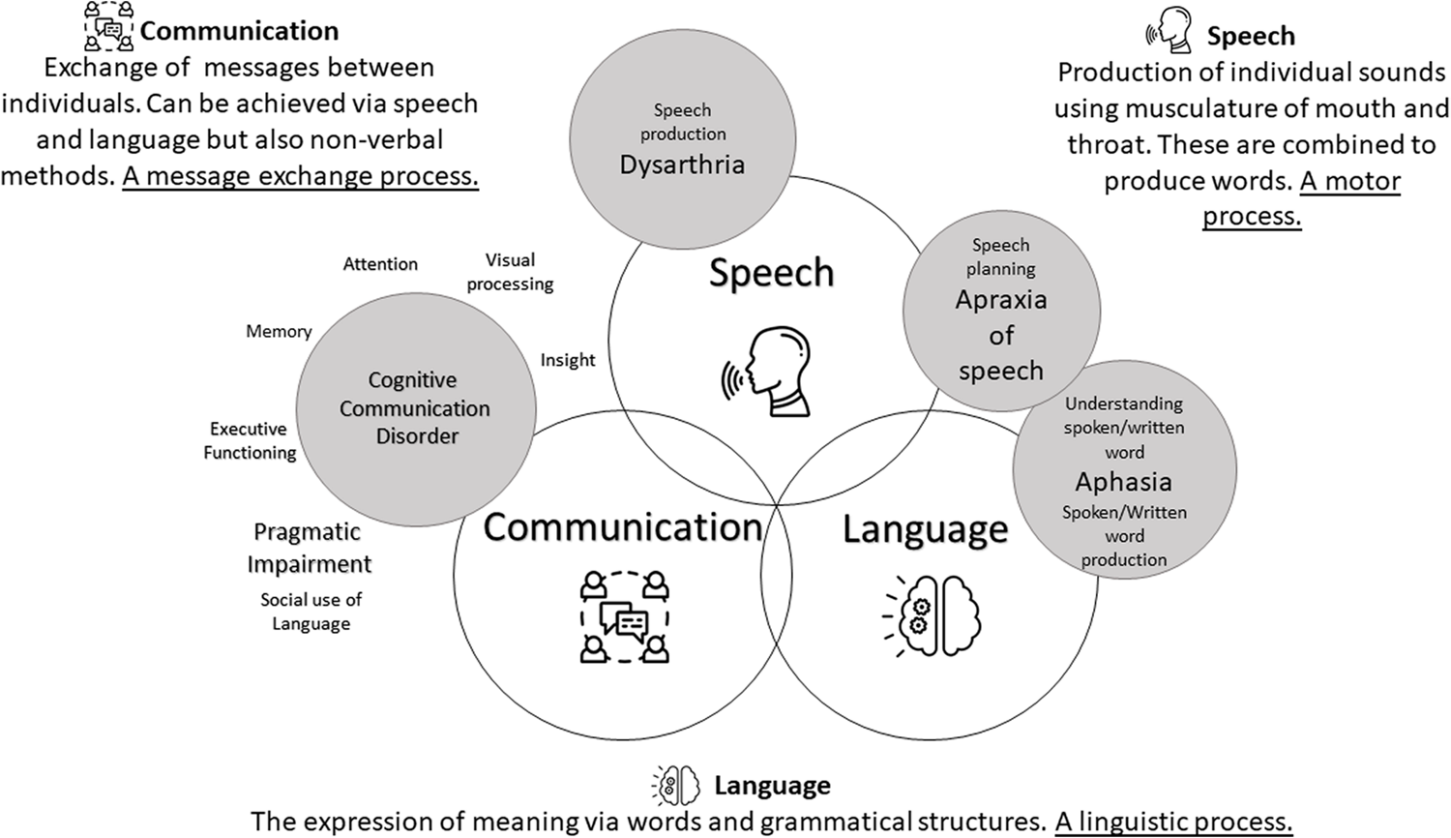
Speech, language, and communication needs in neurological populations

Improved understanding of differences in the range of SLCN after a brain tumour, and more appropriate use of terminology, would be of value. Figure 1 aims to support this by summarising SLCN in adult neurological populations, illustrating how speech, language, and communication are both distinct and closely related. Communication difficulties stemming from speech impairment can be solely related to impaired musculature/motor functioning (dysarthria) or to motor planning (apraxia of speech). These impairments can exist in isolation but may also occur alongside acquired impairment of understanding or production of language (aphasia). There are a broad range of ‘cognitive communication disorders’ where higher level functioning interacts with and influences various aspects of communication such as memory, attention, or social use of language. These types of difficulties can result in features such as repetitive questions, or difficulties staying on topic and in delivering responses at the right time in conversations. Whether speech, language, or communication is impaired (individually or in combination) following a brain tumour, each manifestation will have different consequences and be impacted by personal (e.g., mood/emotional status, resilience, or experience of frustration) or external factors (e.g., support systems, treatment-related fatigue).

### Explore innovative ways to engage with seldom-heard voices

For many who would struggle to take part in large-scale quantitative studies, methodologically rigorous qualitative research offers possibilities for inclusion [16]. There are some examples of this nature in dementia and aphasia research e.g. [17, 18]. Although conventional methods may be challenging (e.g., open questions), data can be collected in creative ways. Experienced facilitators can explore means to convey messages via alternative strategies (e.g., drawing or gesture); ethnography can observe everyday interactions; and conversation analysis can identify sources of communication breakdown. Anyone interested in supporting communication can complete conversation partner training (e.g., the free online course provided by Communication Access UK; https://communication-access.co.uk/). Evidence from aphasia research suggests that improving interactions this way successfully reduces risk of negative psychosocial consequences [19].

### Include and empower speech and language therapists across research

Including speech and language therapists (SLTs) in study teams could support meaningful inclusion in both qualitative and quantitative research. They can advise on alternative and augmentative communication methods, create and facilitate use of accessible study documentation, support mental capacity assessment and the consent process, conduct interviews, and improve accuracy when writing up results. Brain tumour research(ers) can learn from work led and influenced by SLTs, e.g., with populations that present similar challenges [20], while the SLT profession could become more involved as advocates for a vulnerable population.

Discussion of the concerns outlined above and of our suggested ways forward could improve research quality and depth of understanding of the impact of brain tumours on speech, language and communication and QOL. By working together, researchers and clinicians can speak up for the lost voices and generate the evidence to inform the development of services and supports to better meet the needs of those affected by SCLN following a brain tumour.
